# Enabling Virtual AAA Management in SDN-Based IoT Networks †

**DOI:** 10.3390/s19020295

**Published:** 2019-01-12

**Authors:** Alejandro Molina Zarca, Dan Garcia-Carrillo, Jorge Bernal Bernabe, Jordi Ortiz, Rafael Marin-Perez, Antonio Skarmeta

**Affiliations:** 1 Department of Information and Communications Engineering, University of Murcia, 30100 Murcia, Spain; jorgebernal@um.es (J.B.B.); jordi.ortiz@um.es (J.O.); skarmeta@um.es (A.S.); 2 Department of Research and Innovation, Odin Solutions, 30820 Murcia, Spain; dgarcia@odins.es (D.G.-C.); rmarin@odins.es (R.M.-P.)

**Keywords:** IoT, SDN, NFV, channel protection, bootstrapping, AAA, security policies

## Abstract

The increase of Software Defined Networks (SDN) and Network Function Virtualization (NFV) technologies is bringing many security management benefits that can be exploited at the edge of Internet of Things (IoT) networks to deal with cyber-threats. In this sense, this paper presents and evaluates a novel policy-based and cyber-situational awareness security framework for continuous and dynamic management of Authentication, Authorization, Accounting (AAA) as well as Channel Protection virtual security functions in IoT networks enabled with SDN/NFV. The virtual AAA, including network authenticators, are deployed as VNF (Virtual Network Function) dynamically at the edge, in order to enable scalable device’s bootstrapping and managing the access control of IoT devices to the network. In addition, our solution allows distributing dynamically the necessary crypto-keys for IoT Machine to Machine (M2M) communications and deploy virtual Channel-protection proxys as VNFs, with the aim of establishing secure tunnels among IoT devices and services, according to the contextual decisions inferred by the cognitive framework. The solution has been implemented and evaluated, demonstrating its feasibility to manage dynamically AAA and channel protection in SDN/NFV-enabled IoT scenarios.

## 1. Introduction

Edge and fog technologies [1] shift centralized clouds towards the edge with the aim to deliver better throughput, enable enhanced context-specific functionality, and support diverse kinds of communications. They also enable localized functions, such as processing the security in Machine-to-Machine (M2M) communication required in IoT, by exploiting nearby resources [2]. The Fog includes another infrastructural level between edge and cloud in which security functions for IoT devices can be offloaded to their vicinity.

Fog computing can drastically improve network connectivity at the edge by leveraging NFV (Network Function Virtualization) and SDN (Software Defined Networks). NFV presents remarkable advantages for deliver virtual appliances in the edge and remote cloud data centers [3]. Dynamic provisioning of virtual security functions towards the edge of the network enhance scalability, necessary to deal with the huge amount of IoT traffic. At this point, the use of SDN becomes essential in order to reconfigure the network dynamically, providing new networking rules on demand, thereby connect the new virtualized services to the existing architecture, as well as enforcing networking countermeasures, such as firewall rules, to mitigate cyberattacks, e.g., Distributed Denial of Service attacks (DDoS).

In this context, Authentication, Authorization and Accounting (AAA) as well as Channel-Protection Network Security Functions (NSF) can be timely and dynamically deployed and configured at the edge in virtualized and softwarized fog entities, such as cloudlets, and IoT gateways, in order to facilitate the security management in IoT networks. To this aim, new context-aware holistic security solutions are needed to allow the orchestration [4] of NFV managers, SDN controllers and IoT controllers, thereby providing security chaining, as well as dynamic reconfiguration and adaptation of the virtual security appliances.

Furthermore, there is a strong need to define proper, inter-operable and highly-expressive security policy languages and models to empower users and administrators to manage, in a high-level fashion, the overall security and privacy aspects of their Fog and IoT entities across the whole ecosystem. Those policy models could serve as input for framework orchestrators to organize and choreograph the aforementioned security services. Some security policy models [5] and frameworks [6,7] had proposed solutions in the past to manage distributed systems. However, they are not tailored to manage cybersecurity in IoT networks and Mobile Edge Computing scenarios, as presented in this paper.

On the other hand, AAA and Channel protection NSFs have been already successfully studied and addressed in IoT networks [8]. However, those NSFs have not yet properly studied and exploited the advantages IoT networks enabled with NFV/SDN technologies, where cyber-situational awareness and policy-based security frameworks can dynamically react and mitigate cyber-attacks by deploying and configuring timely and wisely, in the proper location, suitable virtual NSFs and security/network configuration rules.

This paper extends our previous conference paper [9] by evaluating a novel policy-aware approach to manage AAA and channel protection in SDN/NFV-enabled IoT networks. Our virtual AAA (vAAA) NSF, including network authenticators, are deployed and activated dynamically at the edge, facilitating the device’s bootstrapping and ruling the access control of IoT devices to the network, by relying on SDN to enforce the network authorization decisions in the switches. Likewise, the proposed scalable channel protection management allows dynamic provisioning the necessary crypto-keys for IoT M2M communications, establishing Datagram Transport Layer Security (DTLS) channels among IoT devices and services. The process is driven in a centralized way by the Security Orchestrator, adopting an scalable, softwarized and cyber-situational awareness [10] approach, which enables key-management and the enforcement of security association in both sides of the protected channel. Unlike in our previous conference’s paper, this paper has improved the original design, implemented the solution, and evaluated the feasibility and performance of our proposed SDN-based AAA and channel-protection solution for IoT.

The rest of the paper is organized as follows. Section 2 analyzes current state-of-the-art about security solutions for IoT systems based on NFV/SDN. Section 3 overviews the cyber-security and policy-based framework and its applicability to deal with AAA and Channel protection. Section 4 delves into the proposed vAAA NSF for IoT. Section 5 is devoted to the softwarized IoT Channel Protection proposal. Section 6 is a promising use case is presented to assess the introduced security features and performance evaluation is carried out. Finally, conclusions and ongoing research are drawn in Section 7.

## 2. Related Work

Large scale IoT deployments [11] are comprised of diverse devices that implement different protocol stacks. In this context, providing an inter-operable and open bootstrapping solution will ease the deployment of the different devices of an IoT network. In this sense, to the best of our knowledge, this work is the first attempt to integrate NFV and SDN management of IoT bootstrapping for large deployments with AAA federation support, which is compatible with diverse bootstrapping solutions. We focus on the use of AAA because, in general, solutions that aim to provide a scalable secure bootstrapping solution for IoT use Extensible Authentication Protocol (EAP) and AAA [12].

The Zigbee IP [13] standard is one of the first complete solutions for IoT. It uses the Protocol for Carrying Authentication for Network Access (PANA) and EAP for network access authentication. However, AAA is not considered in the standard; they use the standalone mode and fix the EAP method to be used to EAP-TLS which limits the flexibility offered by EAP. Currently, there is work in standardization organizations such as the Internet Engineering Task Force (IETF) to define new protocols for channel protection and key exchange and distribution in IoT, such as the OSCORE [14] and EDHOC [15] protocols; the former is used to secure the communications end-to-end, while the later generates the necessary key material. Nonetheless, the current standard to protect Constrained Application Protocol (CoAP) exchanges is DTLS. CoAP documentation defines DTLS as its secure communications mechanism. Therefore, DTLS is one of the first protocols to be considered in IoT security associations.

We can distinguish protocols that establish a security association (SAP); protocols that use the Security Association (SA) to protect the channel; and protocols that bootstrap all of the above. In small scale deployments, a simple SAP with the key material set up at both ends of the authentication process normally suffices. For instance, DTLS can be employed to set up the necessary key material to establish the security association between the IoT device and the Gateway, but when large scale deployments and multi-domains are considered, it is interesting to rely on more scalable alternatives such as AAA and EAP to bootstrap the key material in order to establish security associations like the aforementioned DTLS channel. Therefore, our solution leverages EAP to facilitate scalable key management in IoT deployments, and then manages the establishment of DTLS channels to protect IoT communications with such a derived key material.

The SDN has demonstrated to be a flexible and powerful enabler to new network solutions. The centralized control provides complete network information, therefore enhancing control decisions. SDN based solutions endow the architecture with desirable features such as flexibility, dynamism, centralized management and scalability.

Current works like [16] show how the SDN can be addressed in order to mitigate security issues at different layers. Network softwarization plays a key role providing with the desired scalability level to the proposal. In this sense, the IETF is working towards managing IPSec Security Associations (SAs) in SDN networks and enabling end-to-end channel protection [17]. Unlike that interesting initiative, our work intends to get the benefits of SDN to facilitate channel protection in IoT networks in which IPSec is not directly supported, or which just require establishment of additional secure channels using DTLS.

On the other hand, regarding NFV technologies, they avoid the deployment of specific hardware equipment through the use of virtual machines running specific network functions on commodity servers. NFV provides among others, flexible provisioning, deployment and centralized management. The possibility to employ Virtual Network Functions (VNF) to deploy security appliances is an interesting alternative to enhance an architecture with adaptive and reactive security capabilities. Since it is possible to deploy security appliances as virtual network functions (VNF) in a Network Function Virtualization environment, this approach becomes really interesting in order to provide adaptive and reactive security capabilities to an architecture [18].

In this sense, in [19], the authors highlight different benefits of integrating SDN within the NFV, coming up with a software-defined NFV architecture, which allows for taking advantage of both softwarization and virtualization. Similar SDN/NFV approaches are being applied to leverage the security management of IoT scenarios in 5G IoT networks [20]. Regarding policy-based security management, in [21], the authors proposed an approach towards the adoption of security policies management with dynamic network virtualization. In [22], we proposed a preliminary joint use of security policies, SDN and NFV security appliances, focusing the performance on dynamic network filtering. Unlike that work, which focuses on dynamic filtering policies enforcement, this paper takes advantage of policy-based security framework to deal with AAA and channel protection security functions, thereby providing performance evaluation for each policy deployment process—all of this using physical IoT devices.

## 3. Security Management Framework and Proposal Overview

This section overviews the ANASTACIA architecture (ANASTACIA Project [23]) in order to present the main building blocks that have been needed to design and implement our solution.

### 3.1. ANASTACIA Framework Overview

The ANASTACIA framework [24,25] provides a context-aware autonomous security orchestration in SDN/NFV-enabled IoT networks. The framework orchestrates dynamically the network security according to the context obtained from agents, whereby mitigating and countering cybersecurity threats at the edge of the network in IoT scenarios, by deploying and orchestrating Virtual Security Functions and services even over constrained IoT devices. The security framework is endowed with monitoring and reaction tools as well as innovative algorithms and techniques for threat analysis, and correlation from different sources. Thereby, increasing the overall security, including self-repair, self-healing and self-protection capabilities, not only at the core, but also at the edge of the network.

Through the use of networking technologies such as SDN-NFV and intelligent and dynamic security policy enforcement and monitoring methodologies, different virtual security appliances such as vFirewall, vIDS, vAAA, vSwitch/Router, vHoneynet, vVPN are orchestrated dynamically at the network edge.

**Figure 1:**
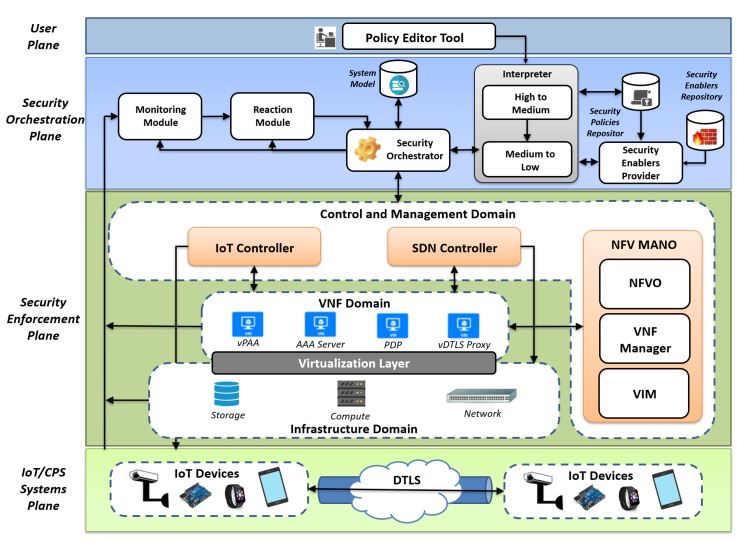
ANASTACIA Framework Architecture overview.

A high level view of the framework is depicted in Figure 1. The **User Plane** includes interfaces, services, and tools to end-users for policy definition, system monitoring and service management. Its policy editor provides an intuitive and user-friendly tool to configure security policies governing the configuration of the system and network, such as authentication, authorization, filtering, channel protection, and forwarding. More detailed information on the framework can be found in its web page and in particular motivation and security analysis regarding ANASTACIA can be found in Public Deliverables 1.2 [26], 2.2 [27] and 2.3 [28], respectively.

The **Security Orchestration plane** enforces policy-based security mechanisms and provides run-time reconfiguration and adaptation of security enablers, thereby providing the framework with intelligent and dynamic behavior. It is an innovative layer of our architecture and provides self-protection and self-healing capabilities for softwarized networks through novel modules. The *Policy Interpreter* module receives as input the policies and identifies the capabilities needed to enforce such policies (capability matching). Then, the Interpreter interacts with the *Security Enablers Provider* to identify the SDN/NFV-based/IoT specific enablers that are able to enforce the desired capabilities. The *Security Orchestrator* selects the enablers to be effectively deployed, accounting for the security requirements, the available resources in the underlying infrastructure, and optimization criteria. The *Monitoring* component collects security-focused real-time information related to the system behavior from physical/virtual appliances. Its main objective is to provide alerts for the reaction module in case something is misbehaving. Security probes are deployed in the infrastructure domain to support the monitoring services. Then, the *Reaction* component is in charge of providing appropriate countermeasures, by dynamically defining reconfiguration of the security enablers according to the circumstances. The reaction outcomes are then analyzed by the Security Orchestrator, which enforce the corresponding enablers’ countermeasures.

The **Control and management domain** modules supervise the usage of resources and run-time operations of security enablers deployed over software-based and IoT networks. A set of distributed SDN controllers takes charge of communicating with the SDN-based network elements to manage connectivity in the underneath virtual and physical infrastructure. ETSI’s NFV-MANO (Management and Network Orchestration)-compliant modules supports secure placement and management of virtual security functions over the virtualized infrastructure. The IoT Controller is intended to manage IoT devices and networks, such as LoWPANs (Low-Power Wireless Personal Area Networks) and LPWAN (Low-power Wide-Area Network).

**Infrastructure and Virtualization domain** This domain comprises all the physical machines capable of providing computing, storage, and networking capabilities to build an Infrastructures as a Service (IaaS) layer by leveraging appropriate virtualization technologies. This plane also includes the network elements responsible for traffic forwarding, following the rules of SDN controllers, and a distributed set of security probes for data collection to support the monitoring services. The **VNF domain** accounts for the VNFs deployed over the virtualization infrastructure to enforce security within network services. It provides advanced security VNFs (such as virtual AAA, and Channel protection used in our proposal), capable of providing the defense mechanisms and threat countermeasures requested by security policies. The **IoT domain** comprises the IoT devices to be controlled. This includes the security enablers, actuators or software agents needed to enforce the security directives coming from the orchestration plane and managed, at the enforcement plane, by the IoT controller.

### 3.2. Solution Overview

**Figure 2:**
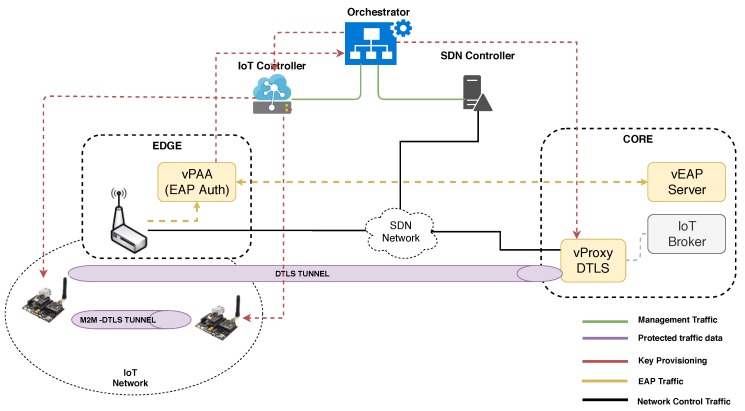
vAAA and vChannel-Protection deployment in the IoT network.

The solution relies on the ANASTACIA framework architecture shown in Section 3.1, improving it to support AAA, bootstrapping and channel protection mechanisms for SDN/NFV-based IoT networks. Figure 2 depicts a general deployment overview of our SDN/NFV-based virtual AAA (vAAA) and virtual Channel-Protection (vChannel-Protection) solution for IoT networks, which allows dynamic management of authentication, authorization, bootstrapping, key management, and channel protection configuration.

As it can be seen in the figure, some of the components such as the virtual EAP (vEAP) Server VNF and virtual Proxy (vProxy) VNF are meant to be deployed in a centralized location, accessible by different IoT networks/domains. On the other hand, other components like the virtual PAA (vPAA) VNF are deployed at the edge of those IoT Networks to facilitate the bootstrapping, authentication and the key management, required to establish secure tunnels.

Namely, the vEAP VNF is responsible for making authorization/authentication decisions, according to the security policies enforced by the ANASTACIA framework through the Orchestrator. Likewise, the vProxy VNF is in charge of managing the channel protection set-up, in the other side of the channel, abstracting end-point services from this task. Thus, vProxy can be deployed and configured dynamically, on demand, by the Orchestrator to facilitate the security association set-up for those entities (like for instance IoT context brokers or cloud-data repositories), which are not aware and capable of establishing DTLS tunnels.

The SDN Controller, as demanded by the security Orchestrator, manages the network communications between the different components, enforcing, through the southbound Application Programming Interface (API), flow rules to drop, filter, allow or redirect traffic, from and towards the IoT devices. Additionally, the IoT controller intermediates to perform key provisioning to the IoT devices, according to the Orchestrator commands. In addition, although it is not shown in the figure for the sake of simplicity, those VNFs are deployed by the NFV-MANO, whenever decided and commanded by the Security Orchestrator. This solution assumes that the network has at least one SDN capable switch located on the edge of the IoT network in order to be able to enforce traffic rules dynamically.

The following sections describe in deep our SDN/NFV-based solution for vAAA and vChannel protection management in IoT networks.

## 4. vAAA in SDN/NFV Enabled IoT Networks

Security has evolved from a desired feature to a requirement to be provided by networking infrastructures. In order to provide a certain level of security, cryptographic material needs to be deployed to the elements involved in the communication. To that end, AAA solutions have been used historically and a whole area of research has been developed around them. This work aims not only to face the AAA architecture virtualization as a network function (NFV) triggered by security or business originated policies challenge, but also taking into account the precise considerations needed to enable these kind of solutions into IoT environments.

### 4.1. AAA Preliminaries

The Authentication, Authorization and Accounting (AAA) framework is used and instantiated, typically, in protocols such as RADIUS [29] and Diameter [30] that give support to a great number of devices. Examples of this are the Eduroam network, or TELCOS mobile deployments. They are used to authenticate the devices, authorize access to the services offered (e.g., Access to the Internet) and keep track of the use. Advanced features such as federation (e.g., exemplified in Eduroam) bring scalability to the deployment of a great number of devices that may belong to different organizations under deployment infrastructures of different operators. The Extensible Authentication Protocol (EAP) is a protocol that offers a myriad of authentication methods, as well as a Key Management Framework (EAP-KMF [31]) that enables the bootstrapping of different Unicast or Multicast security association protocols (e.g., DTLS) to secure the communications. EAP lower layers, such as PANA [32] or Low-Overhead CoAP-EAP (LO-CoAP-EAP) [33], transport EAP between a device and the domain controller to authenticate and provide access to the different services of the domain.

### 4.2. Policy-Based AAA Management

Security management through policies endow administrators with abstraction capabilities of the underlying systems, enabling the definition of high-level security intents independent from the low configurations, which becomes a powerful tool for achieving interoperability and scalability. A policy framework can even provide different abstraction levels through policy refinement, facilitating the interaction with the user based on the later knowledge level. In this way, two users with different level of knowledge could model the same security policy at different levels (e.g., high and medium levels) obtaining the same effect. Likewise, medium level policies can be translated to vendor-specific technical configuration over the system. The policy level separation allows abstracting policy definitions which could potentially be implemented by many different end-points leveraging on different technologies, and therefore making the policies entity and technology agnostic from the underlying system.

In this sense, our solution is based on developed plugins which translate authentication, authorization, and channel protection medium-level security policies into specific device configurations (e.g., vBootstraping VNF, AAA Server, Policy Decision Point, Policy Enforcement Point and so on). The plugin selection decision is made by the Security Orchestrator who has a global cyber-situational awareness [10] vision of the current architecture status (thanks to the monitoring components of the system).

**Figure 3:**
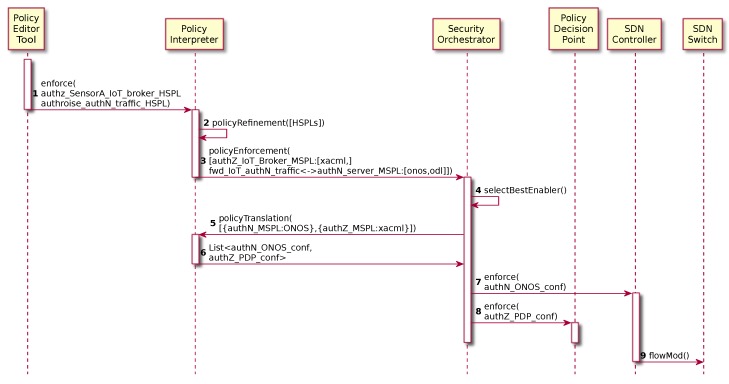
Authentication and Authorization proactive policy enforcement process.

Following this approach, we have extended the ANASTACIA security framework in order to apply security policies in AAA scenarios. To this aim, our proposal extends the security policy models provided by SECURED [7] project (High-level Security Policy and Medium-level Security Policy). Figure 3 shows a proactive high-level security policy enforcement workflow in order to allow the IoT device network authentication as well as authorizing the IoT device to put information in the IoT broker. This is, first, the security administrator models the High-level Security Policies (HSPLs) through the Policy Editor Tool GUI (Figure 3-step 1). These HSPLs are then refined in one or several Medium-level Security Policies (MSPLs) by the Policy Interpreter (Figure 3-step 2). In this case, it generates three MSPLs. The first one authorizes the specified IoT device putting the specified resource in the IoT broker as shown in Listing 1.

Listing 1 **Medium-level Security Policy Language (MSPL) authorization example.**

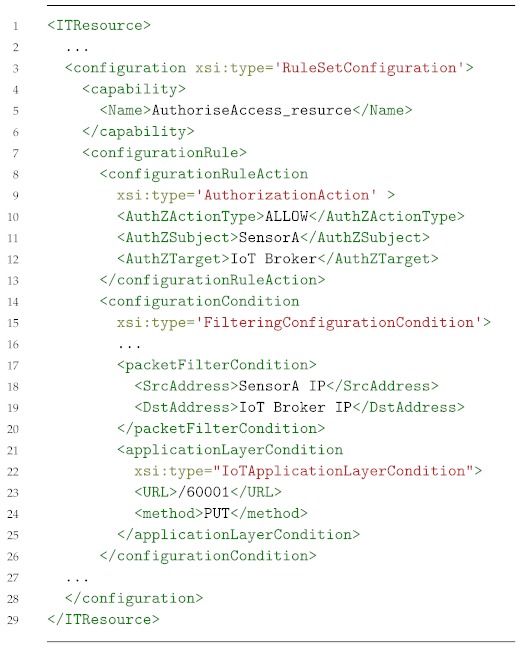

This kind of authorization policy is usually composed of a *subject*, which aims to perform some *action* over a specific target *resource*. In this case, the example is indicating that the *SensorA* (*subject*) is *ALLOWED* to access the resource */60001* using the *PUT* method (*action*) against the *IoT Broker* (*target*). Regarding the other two policies, they allow bidirectional communications, for the authentication protocol, between the IoT device and the network authentication service. Listing 2 shows an example of forwarding policy which indicates that the data from the SensorA with PAA destination and a specific destination port must be forwarded to the PAA through a specified interface. Once the security policies have been refined, the Policy Interpreter requests the MSPLs policy enforcement to the Security Orchestrator, also providing a list of the available security enablers (Figure 3-step 3). Then, the Security Orchestrator analyzes the MSPLs, it selects the best security enabler available which will be able to fulfill the security policy for the underlying infrastructure (Figure 3-step 4) and then it requests the policy translation to the Policy Interpreter in order to obtain the final configurations for each security enabler (Figure 3-step 5). Finally, the Security Orchestrator (SO) receives the configurations (Figure 3-step 6) and it enforces them through the selected security enablers, thereby installing new flow rules in the SDN network allowing the authentication protocol traffic (Figure 3-step 7) and a new eXtensible Access Control Markup language (XACML) authorization policy is installed in the Policy Decision Point (PDP) (Figure 3-step 8).

Listing 2 **MSPL traffic divert example.**

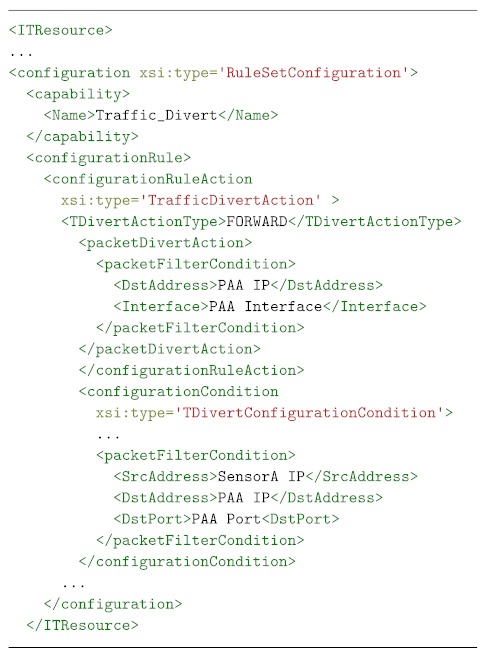

At this point, it is important to highlight that in case the security enabler is not already deployed, the SO will request to a NFV-MANO the deployment of the VNF, and once it has been instantiated, it will be configured with the generated security enabler configuration. To this aim, the SO drives this process and the communication with the NFV-MANO. The SO holds the VNF definitions and NS catalogs, manages workflow of the service deployment and can query the status of already deployed services. The OS interfaces with the Service Orchestrator, and, in turn with the Resource Orchestrator (RO) to provision services over a particular NFV Infrastructure (NFVI) provider (e.g., Openstack) in a given location. Then, the VNFConfiguration and Abstraction (VCA) is contacted by the RO in order to perform the the VNF configuration using Juju Charms. For more information about this process, the reader is referred to our previous work [22].

Once the SDN policies have been enforced, the SDN switch is properly configured in order to allow the authentication traffic among the IoT device and the PAA (Figure 3-step 9).

### 4.3. IoT Bootstrapping

IoT brings heterogeneity of devices and radio technologies, with different capabilities and requirements which have to cooperate and coexist. This paper leverages ANASTACIA framework, providing the design and implementation of different VNFs and security policies needed to deal with bootstrapping AAA and channel protection in IoT. Namely, a VNF is deployed to deal with EAP lower layer (PANA [32] or LO-CoAP-EAP [33] depending on the requirements) for IoT device bootstrapping, authenticate them and manage network access authentication. By having a VNF which deploys an EAP lower layer capable of adapting to each deployment needs, it provides technology flexibility facing of heterogeneity and scalability.

**Figure 4:**
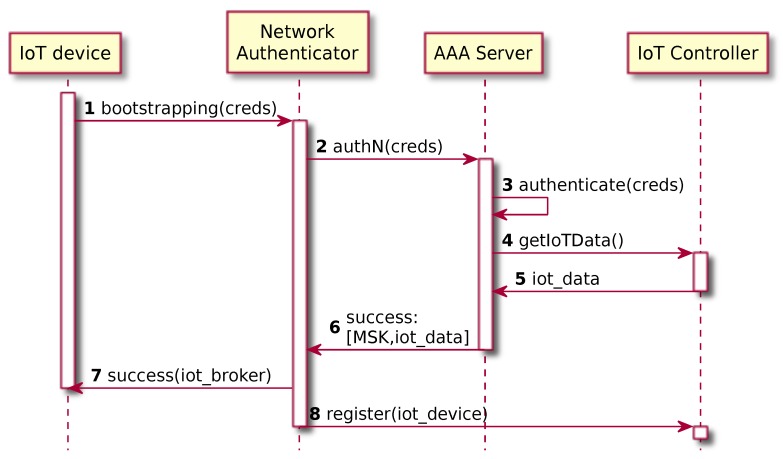
IoT bootstrapping.

Although bootstrapping encompasses several aspects, for the sake of simplicity, this paper focuses mainly on the EAP authentication to provide network access, key derivation and distribution to securely bootstrap other protocols. The proposed bootstrapping process is shown in Figure 4. When the IoT device is turned on, it tries to perform a bootstrapping process against a Network Authenticator IPv6 address provided at commissioning time (Figure 4-step 1). At this point, the Network Authenticator as well as the AAA services may have been instantiated in a proactive approach or as a reaction of the request, and it is important to highlight that the authentication request is able to reach the Network Authenticator since the connectivity was allowed and addressed by the security administrator in Section 4.2.

Regardless the instantiation approach, once the Network Authenticator receives the bootstrapping request, it starts the authentication process against the AAA infrastructure, providing the IoT device credentials (Figure 4-step 2). Then, the AAA Server performs the authentication (Figure 4-step 3), it derives a Master Session Key (MSK) and it also gets relevant information about the IoT device from the IoT Controller such as the default IoT traffic target for the specific IoT device (Figure 4-steps 4,5). Finally, the Network Authenticator obtains the values provided by the AAA Server (Figure 4-step 6), sending the EAP success to the IoT device, including the default traffic target (Figure 4-step 7), and also registering the IoT device to the IoT Controller (Figure 4-step 8).

In the case a device is compromised, thus sending wrong information or a malicious entity planted in the smart building the ANASTACIA framework does the following: In the first place, all traffic not related to bootstrapping is filtered. When the authentication is successful, the traffic according to the permissions of the device is granted. If a device is planted, it will only be able to try to perform the bootstrapping, which will fail due to lack of credentials. If the device is defective, the ANASTACIA framework will point that out, filtering out the traffic and not allowing it to send more information nor to gain access to the network until the device is checked out and repaired.

### 4.4. IoT Device Authorization

**Figure 5:**
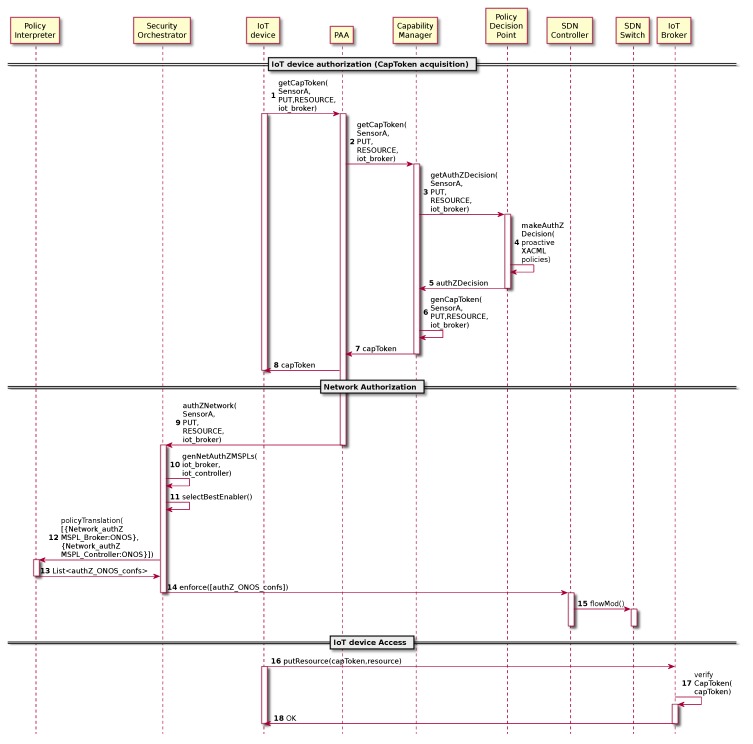
Authorization process.

Once the IoT bootstrapping has been finished, IoT devices must be authorized prior publishing information or accessing any service (e.g., IoT Context Broker). Figure 5 shows the proposed authorization process which uses Distributed Capability-Based Access Control (DCapBAC) [34] as the main authorization approach for constrained devices. That is, the IoT device requests a capability token through the Network Authenticator in order to be able to publish a specific resource value (e.g., temperature) into the IoT broker (Figure 5-step 1). The Ipv6 address of the IoT broker was specified during the bootstrapping process as a default IoT traffic target. The Network Authenticator forwards the request to the Capability Manager (CM) (Figure 5-step 2), who requests the authorization decision to the Policy Decision Point (PDP) (Figure 5-step 3). The PDP verifies whether the specified device is authorized or not to perform the specified operation for thespecified resource and target (Figure 5-step 4). Since the Security Administrator authorized the operation in Section 4.2, the PDP returns a positive authorization to the CM (Figure 5-step 5). Then, the CM generates the capability token for the requested action, valid for a certain time period and signed by itself (Figure 5-step 6) and it sends the result to the Network Authenticator (Figure 5-step 7), which delivers the capability token to the device (Figure 5-step 8) and also requests the network authorization to the Security Orchestrator in order to allow the IoT device reaches the IoT Broker (Figure 5-step 9). It should be noted that the authorization polices, and, in turn, the generated authorization capability tokens can include validity time-period conditions, which reduces the chances of impersonation risks and unauthorized accesses.

At this point, the Security Orchestrator verifies whether there is also some default reaction behaviour for the specified IoT device. For this AAA case, the orchestrator is programmed with an reactive behavior when an IoT device is authorized to use the network, the IoT Controller must be able to reach the aforementioned device, so the Security Orchestrator not only generates a MSPL policy in order to allow the connectivity among the IoT device and the IoT broker, but it also generates a second security policy in order to allow the communication among the IoT Controller and the IoT device (Figure 5-step 10). In this way, the IoT device will be able to receive command and control requests from the IoT Controller.

Once the security policies have been generated, the Security Orchestrator selects the best security enabler which will be in charge of enforcing each security policy (Figure 5-step 11). Since the security policies are networking related and in this case the networking Policy Enforcement Point is a SDN-enabled switch managed by the SDN Controller (ONOS), the Security Orchestrator obtains ONOS networking configurations by the MSPLs policy translation (Figure 5-steps 12,13). Once it receives the SDN configurations, it performs the policy enforcement in the SDN Controller, allowing the aforementioned traffic (Figure 5-steps 14,15). Regarding the IoT device, it tries to put the resource each certain time, but, before doing this, it verifies the validity of the capability token based on a timestamp. If so, the IoT device finally sends the request to the IoT broker Figure 5 -step 16). The IoT Broker then verifies whether the capability token is valid by verifying the timestamp and the signature. Finally, depending on the result, it accepts the resource value or it alerts the unauthorized attempting to the framework Figure 5-steps 17,18). In the specific case where a malicious node is detected either during bootstrapping or during the authorization process (e.g., using invalid credentials), notification of the event is sent to the framework message queue that is processed into the monitoring and reaction modules of the ANASTACIA framework. Then, the alert is evaluated and the framework infer the proper countermeasures to enforce (e.g., apply new filters rules).

Although it is out of the scope of this research, this authorization model can be improved further to consider trust and reputation scores about IoT devices as proposed in [35], or, in our previous work [36], thereby making authorization decisions not only based on authZ policies but also using a trust-aware access control model.

## 5. Channel Protection in Softwarized IoT Networks

### 5.1. Channel Protection

Channel protection has become a main actor in secure communications for guarantying confidentiality and integrity. Nowadays, several techniques are available to protect the communication channel, depending on the Open System Interconnection (OSI) stack level we aim to protect. For instance, at network level, Internet Protocol Security (IPSec) can be applied, while at transport level, depending on the transport protocol used, Transport Layer Security (TLS) or Datagram Transport Layer Security (DTLS) could be employed. The latter guarantees equivalent security levels than TLS but using non connection oriented datagrams as underlying transport.

In the IoT deployment, we use DTLS to protect the communications for the aforementioned reasons and because the current standard protocol to protect the communications defined within CoAP [37] is DTLS. Maintaining the security parameters, credentials and cipher-suites in large deployments can be considered as troublesome. To integrate the channel protection procedure into our framework, we use different VNF such as vBootstrapping, AAA architecture, IoT Controller and IoT Broker to provide security and dynamism into the process, with the use of policies.

### 5.2. Policy-Based DTLS Management in SDN Networks

Similarly to the AAA case, we have followed a policy-based security management approach. A high-level channel protection policy allows for specifying protection requirement regardless of the underlying channel protection techniques and protocols, and the high-level security policy is translated into a medium-level security policy capable of defining more specific securization parameters but still independent from the final implementation.

Listing 3 shows an example of channel protection medium-level security policy. This example aims to provide confidentiality and integrity protection between a DTLS-enabled proxy *(DTLS-Proxy)* and the *IoTDevice* using *AES* as encryption algorithm with a key size of *128* bits in *Counter with CBC-MAC (CCM)* mode. Once the policy has been instantiated, the Security Orchestrator decides the suitable technology to use. In this case, since the devices involved in the securization of the communications are DTLS-enabled (a DTLS-Proxy service is running on the top of the IoT Broker), the Security Orchestrator will choose a plugin in order to translate the DTLS policy to specific configurations of each involved technology, e.g., it generates configurations in order to activate DTLS in the IoT device through the IoT Controller and in the IoT Broker Server. On the other hand, if the device is not DTLS-enabled, a DTLS-Proxy will be deployed at the edge, as close as possible to the mentioned device, and, in that case, the Security Orchestrator will choose a DTLS-proxy plugin as a translator plugin, and it will be also required to apply a forwarding policy in order to collocate the DTLS-Proxy in the path between the two selected devices; ideally, the closer to the non DTLS-enabled device, the better.

Listing 3 **MSPL enabling DTLS example.**

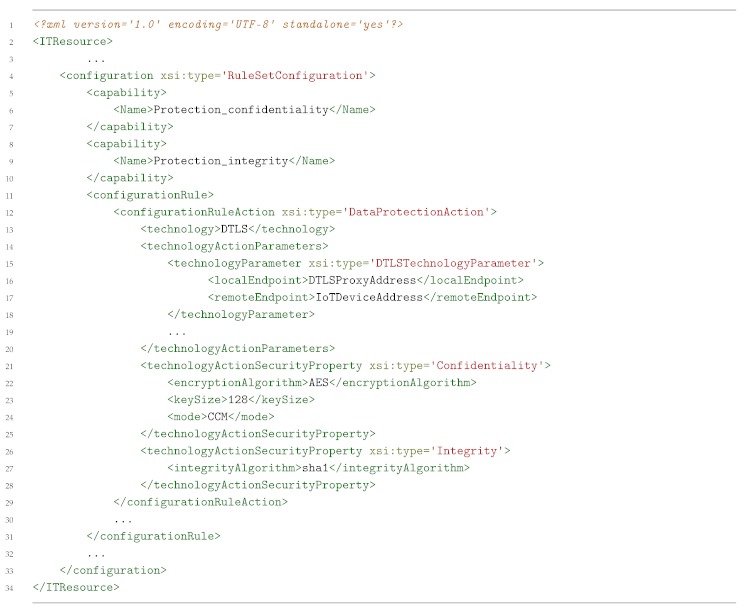

### 5.3. IoT Channel Protection and Key Distribution

**Figure 6:**
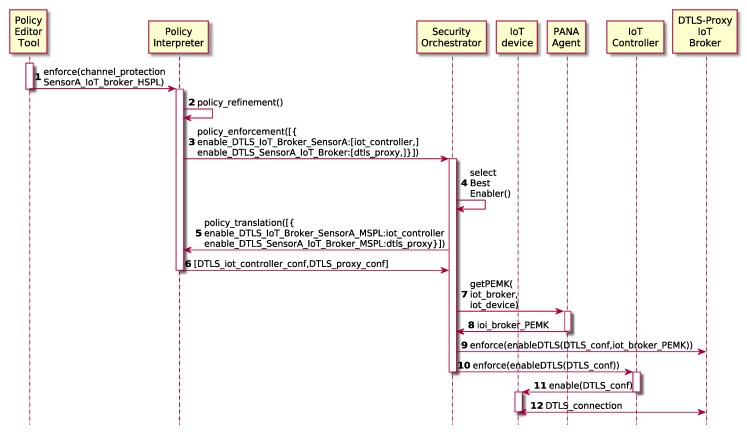
Softwarized and centralized Channel Protection Flow.

Depending on the requirements of the organization such as business strategies, the channel protection could be mandatory or not. If the channel must be always protected, this process can be part of the authorization process, establishing the channel protection policy as another default policy after the authorization as it was shown in Section 4.4. Otherwise, Figure 6 shows the proposed workflow in order to provide IoT end-to-end Channel Protection on demand. The Security Administrator models a high-level security policy through the Policy Editor Tool, indicating that it is necessary to activate channel protection among the IoT device and the IoT broker. The Policy Interpreter performs the policy refinement, transforming the High-level Security Policy (HSPL) in two Medium-level Security Policies (MSPL). After this policy refinement, the Policy Interpreter requests the policy enforcement to the Security Orchestrator, also providing the security enabler candidates, which, in this case, they are the IoT Controller and the DTLS Proxy. The Security Orchestrator decides the best security enabler for each security policy (in this case, we only provide one candidate) and it requests the policy translation to the Policy Interpreter, obtaining the channel protection configurations for the IoT Controller and the Proxy DTLS, respectively (Figure 6-steps 5,6).

Once the Security Orchestrator has received the configurations, it has to enforce them in the security enablers. At this point, it is important to highlight that we have used PANA protocol for carrying the authentication (note that Network Authenticator has been instantiated as PANA Agent), and the PANA Client and Enforcement Point Master Key (PEMK) will be used for the channel protection.

The PEMK is calculated from the MSK, but the IoT device generated the MSK in the bootstrapping process; therefore, the configuration for the other side of the channel (IoT Broker) must be completed with a valid PEMK to establish the tunnel. To this aim, the Security Orchestrator requests the PANA Agent for a specific PEMK to be delivered to the IoT Broker through a secure channel.

The PANA Agent calculates the PEMK (using the MSK it obtained for the IoT device in the bootstrapping process) following pseudo-random function (prf) defined in [38,39] as: PEMK=prf+(MSK,"IETFPEMK"|SID|KID|EPID), where SID is the session identifier, KID is the KEY-ID AVP associated with the MSK and PID is the identifier of the EP.

Then, such a PEMK is provided by PANA Agent to the Security Orchestrator trough a secure channel. At this point the Security Orchestrator has ready the DTLS configurations translated from the security policy, for both sides of the communication channel, i.e., the IoT device and and IoT Broker, so it initiates the process to deliver the configuration to each them.

To this aim, on the one hand, the IoT Controller receives the request from the security orchestrator, which in turn, it generates an IoT protocol specific message (we used CoAP, but it could be any other) with the channel protection configuration to be enforced in the IoT device. The IoT device receives the CoAP message and will try to create a DTLS connection against the IoT Broker using the specific DTLS parameters received. On the other hand, the IoT Broker also receives the DTLS configuration enforcement request from the Security Orchestrator, along with the PEMK required for establishing the connection, and it prepares a DTLS socket, being able to receive DTLS secure connections from the IoT device.

As a result, a softwarized, centralized and dynamic channel protection solution is obtained, leveraging on the authentication process to provide dynamic key management for M2M channel protection, driven by the security orchestrator. In addition, the solution allows for reacting dynamically regenerating and redistributing a new set of keys in case of security breach.

Regarding keys updates, the process is already addressed by PANA. Thus, when the PANA session is close to expire, the PANA Client (PaC) and PAA engage in a re-authentication that has as a result a new EAP exchange, and a new MSK derived from it. As a consequence, the new MSK and all the keys used derived from MSK have to be updated.

It is important to highlight that we considered the end-points are DTLS-enabled, but this is not a mandatory condition. The end-points (including the IoT device) could be DTLS-agnostic. In this case, the framework provides a dynamic DTLS-Proxy VNF. When an end-point requires enabling a channel protection, the Security Orchestrator can request the deployment of a DTLS-Proxy as closer as possible to the end-point, and also request the SDN controller a network configuration in order to redirect the traffic through the new VNF. We want to highlight that the DTLS channel protects the communication between the IoT device and the Proxy-DTLS, the communication between the later and the real end-point might be protected or not, it simplifies the integration of new end-points.

## 6. Proposal Evaluation

### 6.1. Smart Building Use Case

The use case considers an internal attacker performing a sabotage in the building, and the ANASTACIA framework detecting and reacting with the deployment of the softwarized vAAA architecture proposed in this paper.

The actors involved in this use case are the attacker and the security administrator. The attacker is represented by an infected IoT device or a management terminal with a non-trusted operator. On the other hand, the security administrator is the one managing the security policies of the framework.

The triggering of the use case occurs when a terminal within the building network is compromised and starts attacking the infrastructure, thereby the external intrusion defense systems, such as firewalls, are ineffective. At this point, the ANASTACIA framework is able to detect the intrusion and react accordingly.

The ANASTACIA platform, through the Monitoring service and vID, is able to detect an intrusion, firstly analyzing the detected abnormalities and outliers and evaluating the severity of the situation, finally activating prediction mechanisms to ensure that the rest of the building’s system operations continue as expected. Although it is out of the scope of this paper, it is worth mentioning that, in ANASTACIA framework, the attack and intrusion detection can be done using diverse agents and analysis tools, which can employ both signature-based pattern recognition and anomaly-based analysis, according to deviations from the normal behavior of devices monitored by the agents [40].

The platform identifies the attacks and triggers the autonomic self-healing capabilities to deploy dynamically, in the proper location, the vAAA VNF and vBootstrapping VNF and reconfigure the system enforcing the authorization policies in the PDP, and enforcing also, through SDN, in the vSwitch the AC network rules. The vBootstrapping VNF instantiation in our evaluation corresponds to vPAA, but alternate bootstrapping mechanisms might be offered and instantiated based on security or business policies as well as devices’ restrictions.

As a consequence, the devices must be authenticated to gain access to the network. The network is configured to drop any communication from an unauthenticated device, therefore isolating the attacker from the infrastructure. In addition, the communications from trustworthy devices are protected by means of the DTLS tunneling to the vProxy, avoiding traffic inspection and man-in-the-middle attacks among others.

ANASTACIA is currently being validated in a real Smart Building scenario; this paper takes profit from ANASTACIA’s testbed to perform an evaluation of the proposal and integrate the vAAA approach into ANASTACIA’s framework. The deployment used in the testbed follows the overall schema depicted in Figure 2, together with architectural management software components not shown in that figure for the sake of clarity (i.e., Policy Interpreter, Policy Repository, Enabler provider, Capability Manager, PDP).

Regarding the hardware used for the experiment phase, all the components have been deployed in the University of Murcia and they have the following features:

- The Policy Interpreter, Policy Repository, Security Enabler Provider and Security Orchestrator are virtualized and dockerized in an Intel(R) Core(TM) i7-2600 CPU at 3.4 GHz, using three vCores, 3.5 GB of RAM and 30 GB of HDD.
- The IoT Controller is virtualized and dockerized in an Intel Core Processor at 1.5 GHz using 2vCores, 2 GB of RAM and 15 GB of HDD.
- The PAA Network Authenticator, Capability manager, AAA Server, PDP and IoT broker are virtualized and dockerized in an Intel(R) Xeon(R) CPU E5-2603, v3 @ 1.60 GHz with 12 cores and 32 GB RAM and SATA 10k in mode RAID 1 disk drives.
- The SDN Controller is ONOS version 1.15.0.9e4972c5 which has been virtualized and dockerized in an Intel Core Processor (Haswell) at 1.5 GHz using two vCores, 4 GB of RAM and 15 GB of HDD. Control plane is assumed to be isolated from data plane, in this case by means of VLANs.
- The SDN Switch is an HP model 2920, software revision WB.16.04.0008, ROM version WB.16.03.
- The IoT devices are MSP430F5419A-EP at 25 Mhz, 128 KB ROM and 16 KB RAM, running a customized version of Contiki OS 2.7 and erbium CoAP server.
- The 6lowPAN bridge is a MSP430F5419A-EP at 25 Mhz, 128 KB ROM and 16 KB RAM, running a customized version of Contiki OS 2.7 in order to allow the communication between 802.15.4 and 802.3.

Regarding the software used for the experiment phase:

- The PANA authentication software is based on PANATIKI (https://sourceforge.net/projects/panatiki/) implementation for the IoT device, and a modified version of the OpenPANA implementation (https://sourceforge.net/projects/openpana/) for the PAA.
- The distributed authorization token is based on an implementation of the Capability Token [34].
- For the DTLS communication, tinyDTLS (https://sourceforge.net/projects/tinydtls/) is used within the IoT device while Californium (https://github.com/eclipse/californium) is employed within the DTLS proxy in charge of enable the DTLS communication and decrypt the IoT egressed DTLS/CoAP messages to the CoAP required by the IoT broker to publish information.
- PDP, DTLS Proxy and IoT Controller plugins have been implemented from scratch in python.
- AAA policy refinement and translation, IoT registration, IoT Controller, Key management and PDP APIs have been implemented from scratch in python.
- All the elements in the experiment have NTP synchronized avoiding false negative cases due to clock mismatch with the capability Token.

### 6.2. Performance Evaluation

The performance evaluation of the proposed security solution within the ANASTACIA framework implied the implementation of new plugins and the security enablers associated with them: the XACML, DTLS proxy and DTLS IoT.

Similarly, the endpoints and APIs that allow the enforcement procedures from the Security Orchestrator employing these new Security Enablers have been implemented.

There are three processes that involve a secured communication between IoT devices and the broker: Authentication, Authorization and Channel protection. Each process consist of four different phases: policies refinement, translation and enforcement within the architecture in one hand and IoT devices’ actions in the other.

The IoT actions corresponding to each process are *bootstrapping* as the Authentication process, *Capability Token retrieval* as the Authorization process and *Handshake* as the channel protection mechanism, since the Handshake is considered the most expensive process during the data push from the IoT device.

**Figure 7:**
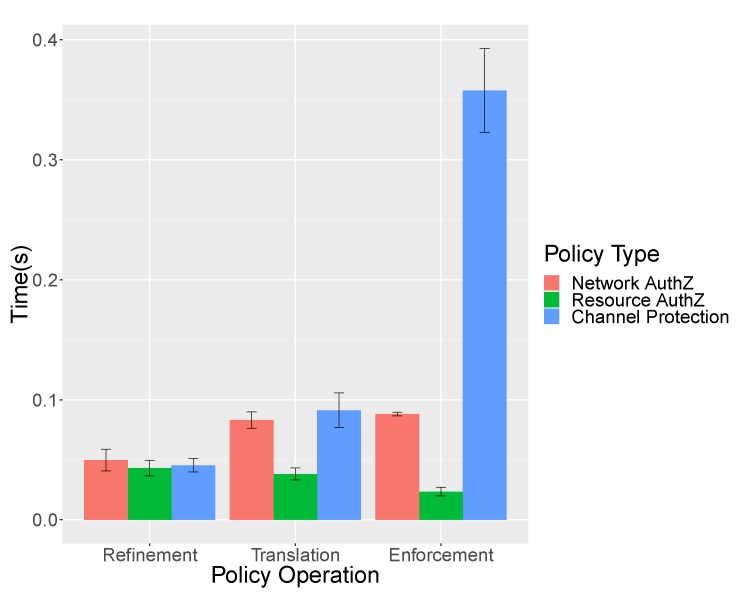
Mean time of policy operation per policy type.

Figure 7 shows the measurements for the policy refinement, translation and enforcement operations for the three processes: network authorization, resource authorization and channel protection, respectively.

As we can see, in the Policy Refinement phase, the network authorization is the most expensive process because it generates two different medium level security policies to allow bidirectional traffic for the authentication (0.74% of the total time). For the resource authorization, only one policy is generated (0.64% of the total time). Lastly, for the channel protection, a medium-level policy is generated and replicated (0.68% of the total time), therefore reducing the time consumption in comparison with network authorization process where two complete policies need to be generated.

During the Policy Translation phase, the network authentication policies generate two different SDN rules for the same technology (1.24% of the total time) and the resource authorization policy only is translated in an XACML policy for the PDP (0.57% of the total time); meanwhile, the channel protection security policies generate two different configurations, for the proxy DTLS and for the IoT Controller (1.37% of the total time).

The Policy Enforcement phase implies the communication with the SDN controller to add the SDN rules that will later be installed by the former into the devices (1.32% of the total time) that will enable the Authentication, followed by the Authorization performed by simply installing the XACML policy into the PDP (0.35% of the local time). Finally, as part of the channel protection phase Policy Enforcement process (5.38% of the total time), the PEMK for the IoT, DTLS Proxy pair is retrieved by the Security Orchestrator. The PEMK with the needed configuration is then provided to the DTLS Proxy that will prepare a DTLS socket with the provided configuration (43% of this phase time) and also provided in parallel to the IoT controller that provides the cryptographic material in addition to the configuration via CoAP message through the IoT network (implying 57% of this phase time).

**Figure 8:**
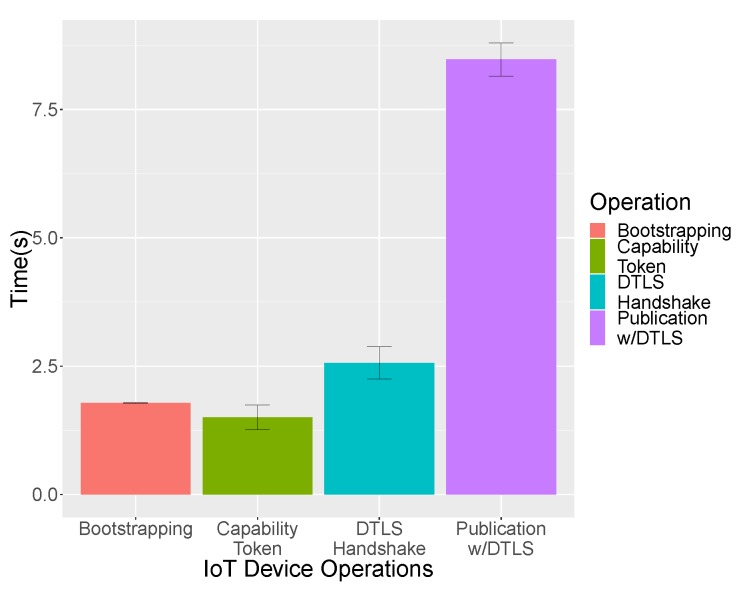
Mean time of each IoT operation (AuthN, AuthZ, Channel Protection).

The evaluation of the solution from the perspective of the IoT devices is shown graphically in Figure 8 and complemented by Table 1. The operations in sequential order are: Bootstrapping done using the PANA protocol, obtain the distributed authorization token and finally establish the CoAP connection to the proxy, which, in turn, communicates with the IoT Broker via HTTP (since fiware does not support CoAP; otherwise, CoAP would have been used), which we have split into handshake and the data publication. Each operation has been carried out 30 times in order to obtain statistically meaningful results.

We can appreciate by observing the values in Table 1 that the time each operation takes to be completed is directly proportional to the number of bytes and messages sent over the network. The relevance of the obtained results is manifested by the fact that, using real hardware, they are consistent with those obtained in previous research [12] based on simulations, in particular based on the Cooja Simulator [41] for the Contiki operative system.

The first row of Table 1 shows the number of messages and total number of bytes exchanged for the PANA Bootstrapping. This instance represents a complete exchange using the EAP-PSK method. The next step, getting the Capability Token is done with the PANA for dynamic credential provisioning extension [42]. The DTLS handshake in PSK mode is done next. The publication using DTLS to protect the CoAP exchange is fragmented at application layer using the CoAP block option, fragmenting the message for publication containing the Capability Token.

Table 2 shows the time measurements for each process. The authentication process implies the time since the security administrator enables the authentication in the front-end, until the IoT devices performs the bootstrapping phase. For the authorization, the time since the administrator enables the access to the resource until the IoT device retrieves the capability token is measured. Finally, the channel protection process involves the time employed since the activation of DTLS channel protection by the administrator to the IoT device DTLS handshake finalization. As can be seen, the solution produces a time overload around 12% per operation. It means that an IoT device consumes around seven seconds from boot until the channel to the IoT broker is protected, of which around one second corresponds to framework processing.

The scalability evaluation has been focused on the DTLS management, from the point in which the security administrator requests the policy deployment (which implies refinement, translation and enforcement) until the DTLS Proxy receives the DTLS configuration and the IoT controller sends the DTLS configuration to the IoT devices. It is worth mentioning that DTLS configuration provisioning to the IoT devices is detached from this process using non-confirmable CoAP messages; the IoT controller will then attend each device independently depending on specific factors such as the availability or the network load.

**Figure 9:**
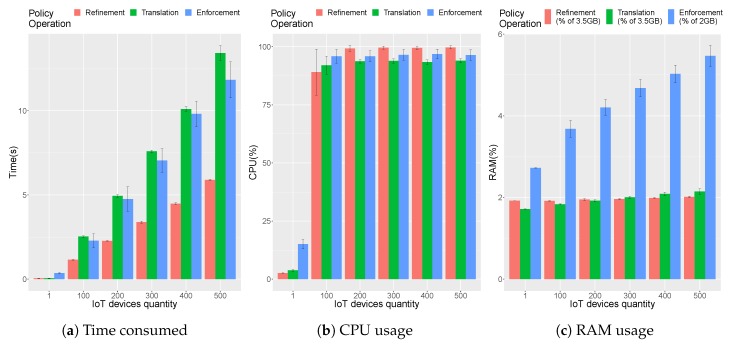
Scalability evaluation.

Figure 9 shows the results for 30 use case executions for deployments running from 1 to 500 IoT devices. Since the real testbed is composed by less IoT devices, multiple non-confirmable CoAP messages were issued for each available IoT device until completing the total amount of desired IoT devices.

As can easily be seen, for one IoT device, the policy enforcement is more expensive than the refinement and translation processes since the processing of one security policy is lighter than the PEMK calculation, distribution and the IoT configuration through the IoT network. When the number of IoT devices increases, the most expensive process becomes the policy translation (around 43% of the DTLS policy deployment time for 100 devices); this process translates two completely different MSPL policies per DTLS configuration into DTLS Proxy configurations and into IoT Controller DTLS configurations. The following process in terms of time consumption is the policy enforcement (around 38% of the DTLS policy deployment time for 100 devices) in which the DTLS Proxy is configured while the IoT Controller launches a thread for each IoT device configuration. The rest of the deployment time is spent by the refinement process (around 19% of the DTLS policy deployment time for 100 devices), which generates the same MSPL twice, one for each target of the DTLS connection.

It is important to highlight that Figure 9a blue enforcement bars represent the aggregation of IoT device configuration by the IoT Controller with the Proxy DTLS enforcement times. In this case, while for one IoT device, the IoT Controller enforcement took the 57% of the enforcement process, as the IoT device number increases, the difference among the Proxy DTLS enforcement and the IoT Controller enforcement also increases. In particular, for 500 IoT devices, the time spent by the IoT controller becomes 97.73% of the whole enforcement process time.

As a global view, the biggest test, with 500 IoT device DTLS policies deployed (refinement + translation + enforcement) took around 30 seconds. The data show that the solution clearly provides a linear deployment time increase trend related to the number of IoT devices being secured.

Figure 9b shows how the CPU stays at 100% except on the base case with one device. This is why time in Figure 9a is increasing in relation to the amount of IoT devices. At the same time, memory usage is stable for the refinement and translations processes as shown in Figure 9c, while it is increasing with the number of IoT devices for the Enforcement, which is related to the fact that the CoAP messages need to be sent over the IoT network; therefore, queuing is needed and memory is therefore reserved longer nevertheless not exceeding 6%≈120MB of the devoted total amount of RAM.

Regarding the experimental replicability of the obtained results, similar results should be achieved when similar hardware is employed. That is, the more constrained equipment and IoT devices, the worse result in the performance. Furthermore, the radio technologies used are also a factor in the performance. We consider that this deployment is an instantiation of the use case that provides a reference by using affordable hardware (the newest CPU is five years old) and software, so enhancing the CPUs employed would clearly lead to enhanced results.

## 7. Conclusions

This paper has evaluated a novel on-demand virtualized AAA and an associated channel protection mechanism specially designed to work on IoT deployments and orchestrated by a wider security architecture, the ANASTACIA framework.

The paper has described how the vAAA can bootstrap an IoT device and distribute the encryption material to the involved network elements and how DTLS channel protection is achieved. In addition, sample authorization and channel protection policies describing the aforementioned mechanisms have been proposed and instantiated by implementing the refinement process as well as the proper translation plugins. Finally, it has been also provided a performance evaluation of the solution for the authentication, authorization and channel protection processes in a testbed that mimics a real scenario, comparing the results with previous research based on simulation. The evaluation serves also as a demonstration of the feasibility in terms of the time employed by an IoT device to join a secure IoT deployment. Special focus have been put on the scalability of the system evaluating an IoT network integrated by up to 500 IoT devices. The results show that complex security policy operation like the DTLS enforcement for 500 IoT devices is achieved in around 30 s, which is an affordable response time in the most common IoT deployments. An overload of 12% of the time per operation is a low price to pay taking into account the solution capabilities.

Along with the experiments, we also identified interesting new challenges that we will consider as future work, such as using different authentication protocols on demand in a transparent way for the final devices, or extending the IoT controller functionality in order to enforce an adaptive behaviour based on the contextual inferred decision taken by the cognitive framework. In addition, as future work, we envisage managing AAA and Channel protection security functions in 5G-enabled IoT networks (NB-IoT) to enable end-to-end network traffic isolation and slicing.

## Figures and Tables

**Table 1:** Number of exchanges and bytes per IoT device operation.

IoT Device Operation	Message Count	Total Bytes	μ s	δ s
Bootstrapping w/PANA	11	636	1.7816	0.0059
Getting Cap Token w/PANA [42]	2	836	1.5058	0.2418
DTLS Handshake	9	1200	2.5634	0.3167
Publishing information w/DTLS	24	3081	8.4733	0.3254

**Table 2:** Measurements by process.

Process	Policy Refinement	Policy Translation	Policy Enforcement	IoT Actuation	Total (s)
AuthN	0.049	0.082	0.087	1.781 (Bootstrapping)	**1.999**
AuthZ	0.043	0.038	0.023	1.505 (CapToken)	**1.609**
Channel Prot.	0.045	0.091	0.357	2.544 (Handshake)	**3.037**
**Total (s)**	**0.137**	**0.211**	**0.467**	**5.83**	**6.645**
